# United by Hope, Divided by Access: Country Mapping of COVID-19 Information Accessibility and Its Consequences on Pandemic Eradication

**DOI:** 10.3389/fmed.2020.618337

**Published:** 2021-01-27

**Authors:** Amiel A. Dror, Nicole G. Morozov, Eli Layous, Matti Mizrachi, Amani Daoud, Netanel Eisenbach, Doaa Rayan, Edward Kaykov, Hesham Marei, Masad Barhum, Samer Srouji, Karen B. Avraham, Eyal Sela

**Affiliations:** ^1^Department of Otolaryngology, Head and Neck Surgery, Galilee Medical Center, Nahariya, Israel; ^2^Azrieli Faculty of Medicine, Bar-Ilan University, Safed, Israel; ^3^Sackler Faculty of Medicine, Tel Aviv University, Tel Aviv, Israel; ^4^Geriatric Medicine Department, Galilee Medical Center, Nahariya, Israel; ^5^College of Dentistry, Gulf Medical University (GMU), Ajman, United Arab Emirates; ^6^Oral and Maxillofacial Department, Galilee Medical Center, Nahariya, Israel; ^7^Department of Human Molecular Genetics and Biochemistry, Sackler Faculty of Medicine and Sagol School of Neuroscience, Tel Aviv University Tel Aviv, Israel

**Keywords:** accessibility, COVID-19, disabilility, information accessibility, global health, disability accessibility, website accessibility

## Abstract

Many government websites and mobile content are inaccessible for people with vision, hearing, cognitive, and motor impairments. The COVID-19 pandemic highlighted these disparities when health authority website information, critical in providing resources for curbing the spread of the virus, remained inaccessible for numerous disabled populations. The Web Content Accessibility Guidelines provide comparatively universally accepted guidelines for website accessibility. We utilized these parameters to examine the number of countries with or without accessible health authority websites. The resulting data indicate a dearth of countries with websites accessible for persons with disabilities. Methods of information dissemination must take into consideration individuals with disabilities, particularly in times of global health crises.

## Introduction

The COVID-19 pandemic is challenging the boundaries of not only social behaviors and cultural institutions, but also the rapid and accurate dissemination of information. The containment of this epidemic has required stringent adherence to interpersonal behavioral modifications which are often developed and transmitted by national health authorities. Médecins Sans Frontières advocates for inclusive COVID-19 outreach and educational campaigns with the necessary accommodations specifically for people with disabilities (1). However, national health authority websites may lack website accommodations for people with vision, hearing, physical, or cognitive impairments. Because the COVID-19 pandemic has uniquely impacted communities affected by visual, hearing, cognitive, and motor impairments, minimizing the information gap between persons with and without disabilities is imperative for achieving global engagement in containing not only COVID-19, but also future pandemics (2). We sought to determine what percentage of national health authority websites are fully accessible to people with disabilities according to Web Content Accessibility (WCAG 2.1) guidelines benchmarks (3). Our research demonstrates that only a small percentage of government health websites are fully accessible for people with disabilities.

According to the World Health Organization (WHO), an estimated 2.2 billion people suffer from vision impairment or blindness, while 466 million people have a disabling hearing loss (4, 5). Individuals with temporary or permanent motor or cognitive impairments also require accessibility modifications for proper interaction with websites. Inconsistent heading level and font size or color contrast of elements in webpages harbor barriers for proper interaction by visually impaired people. Likewise, alternative textual descriptions of visual elements on a page are essential for contextual understanding, in addition to proper interaction with text-to-speech engines. Lack of video content subtitles or transcripts present barriers to the hearing impaired. Compatibility with keyboard navigation, including skip linking in the backend of a website, is crucial to accommodate web navigation for people with motor impairment who interact with a single finger or with other motor gestures.

The Web Accessibility Initiative (WAI), launched and endorsed by the World Wide Web Consortium (W3C) (6), established a set of guidelines according to four accessibility principles: whether the website is Perceivable, Operable, Understandable, and Robust. An example for “perceivability” is whether a graphical table on a web page is able to be presented auditorily or via another method for a user while an example for “understandability” is whether a document contains a list of acronyms or initialisms to help the reader understand the abbreviations within the text. In this report, we used WAI guidelines to examine the accessibility of health authority websites worldwide.

## Materials and Methods

Each WCAG 2.1 principle has a set of testable criteria with a total number of 78 testable success criteria. Each success criteria is assigned to one of three conformance levels: A (lowest), AA (intermediate), and AAA (highest). The adherence to higher levels of conformance has been shown to improve accessibility for users with and without disabilities (7).

A panoply of web accessibility evaluation plug-ins was developed under open-source license for the systematic evaluation of website accessibility against the WCAG 2.1 criteria (3). A list of available tools are presented by the W3C website without an official recommendation for usage of one tool above another (8). These automated tools aim to complement the cardinal manual check of a website during the development process and throughout routine website updates to ensure maximal adherence to WCAG guidelines (8, 9). A comprehensive comparison between eight widely used accessibility evaluation tools highlights the strengths and weaknesses of each tool and recommends using more than one tool for optimal coverage of success criteria (10). In other words, while manual checks of websites by people can determine the usability of the website, automated applications can streamline the process and find hidden accessibility pitfalls within the webpages.

Hence, to test the accessibility of COVID-19 information disseminated through health authority websites, we utilized two independent accessibility evaluation engines including WAVE chrome extension (wave.webaim.org) and Accessibility Insights (accessibilityinsights.io), both of which have been described and utilized in previous literature (10, 11). The WAVE tool analyzes 180 checks according to two conformances level (152 level A; 28 level AA); whereas the Accessibility Insights tool analyzes 64 checks according to three conformances level (55 level A; 7 level AA; and 2 level AAA) (9). It must be noted that the weight of each error (e.g., minor, moderate, critical) is defined by the tool developer and thus may result in different impacts on the overall accessibility rank of the page results (10).

Due to the rapid growth of COVID-19 information and the frequent updates of health authorities' websites, which may influence the accessibility score at a given time point, the degree of accessibility of each website was evaluated at three different time points and the presented data refer to the following three consecutive days (5–7 April, 2020). The calculated number of errors of each health authority homepage augments the average number of errors in each test separately (WAVE and Accessibility Insights), with removal of redundant errors represented in both tests.

In addition to accessibility assessments, we tested each website for mobile usability in concordance to Google webmaster developer tools (developers.google.com). In this regard, previous studies have demonstrated that mobile-friendliness of a given website contributes not only to end user usability, but also for website visibility on search engine results (11, 12).

The list of health authorities' websites of 189 countries were drawn from The Geneva Foundation for Medical Education and Research (GFMER) (Supplementary Table 1) (13). Prior to accessibility evaluation, a manual check of each website on the list yielded 174 health authority websites. Websites of 15 countries were excluded due to an inability to load the site on the test server or when the official health authority homepage appeared as a social media page. This was a cross-sectional study concentrating on the accessibility of health authorities' websites' homepages (unit of analysis) providing health information and recommended public protective measures against COVID-19.

## Results

**Figure 1:**
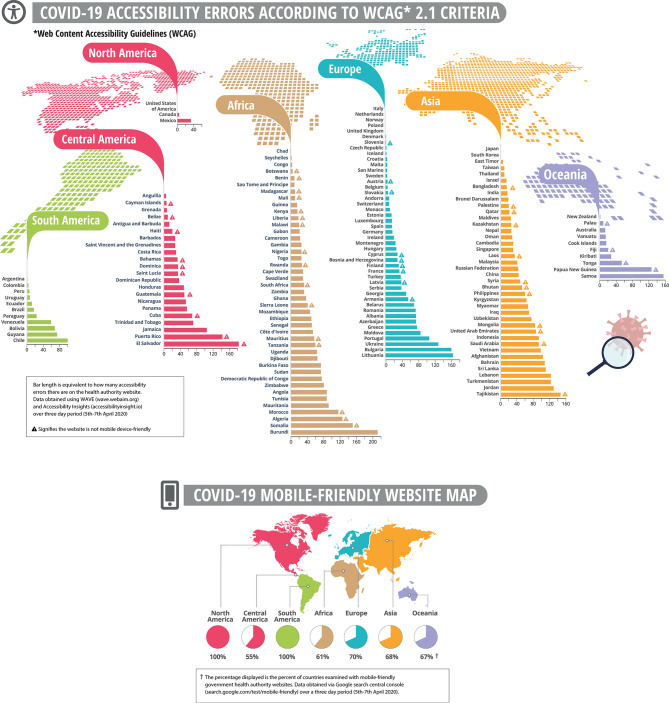
Health authority websites of 174 countries worldwide, demonstrating accessibility errors and mobile friendly maps. The calculated number of errors of each health authority website augment the number of error results in each accessibility evaluation tool separately (WAVE and Accessibility Insights), following removal of redundant errors that are represented in both tests. Mobile computability check according to the Google web developer tool with either pass or fail results. All tests were performed on three consecutive days (5–7 April 2020).

Only 4.7% of the countries examined had fully implemented the WAI accessibility guidelines: Italy, the Netherlands, Norway, Japan, Poland, South Korea, the United Kingdom, and the United States (Figure 1). In contrast, sites from the majority of countries continue to have accessibility errors that present significant barriers to people with disabilities around the world. Distribution of reported errors across all 174 tested health authorities' homepages, according to WCAG conformance levels, reveals that 89% violate Level A criteria, while 11% of countries contain errors that violate higher levels of success criteria (AA and AAA). Inspection of the numbers of errors on all tested pages grouped by WCAG principles indicate that the most impacted principles are robustness (39%) and perceptibility (32%), as compared to operability (19%) and understandability (10%). While both error number, conformance, and principle distribution may be altered according to the selected assessment tools, the data collected signifies the insufficient implementation of WCAG guidelines in the majority of health authority websites, rendering accessibility barriers to millions of people.

## Discussion

Reducing transmission of SARS-CoV-2 depends on tight adherence of the public to simple but challenging modifications in social and public behavior (14). Digital media provide numerous platforms to distribute essential information to the public through websites, social media, and instant messaging applications (15).

Due to the diversity of reporting sources and the harmful consequences of disinformation, governments often encourage the public to check local health authority websites frequently for regular updates (16). This demand requires the information on official websites to be accessible to as many citizens as possible. Unfortunately, individuals with the greatest need for timely and precise data may have the most difficulty accessing governmental material (17). Providing consistently high-quality government productions could also lead to a greater utilization of the Internet by persons with disabilities. Enhancing accessibility to government-sponsored resources could lead not only to immediate population benefits but could also promote the position of people with disabilities in the digital sphere through increased communication, global engagement, and visibility.

Despite remarkable technological advancements in recent history, for people with visual, hearing, motor and cognitive impairments, a seemingly simple website interaction can present a daunting challenge. Although internet access is still unavailable to approximately one-third of the world's population, the needs of all existing users must be accommodated to ensure equal benefits and access to essential health information. The growth and expansion of the Internet must therefore be accompanied by an equal development of sophisticated accessibility technologies, which would expand the usability of the web to individuals with disabilities. With over 2.2 billion people, worldwide, living with vision impairments, an undeniably large section of our society requires accommodations for regular interactions with digital media (3). Beyond the practical benefits of enhanced accessibility, promoting inclusivity for persons with disabilities contributes to an egalitarian society. Without underestimating the importance of accessibility implementation during normal times, the current COVID-19 pandemic now highlights just how important unhindered access to government websites is during a global health crisis.

## Data Availability Statement

The original contributions presented in the study are included in the article/Supplementary Material, further inquiries can be directed to the corresponding author/s.

## Author Contributions

All authors contributed to the production of this manuscript and have approved the final version.

## Conflict of Interest

The authors declare that the research was conducted in the absence of any commercial or financial relationships that could be construed as a potential conflict of interest.
